# New Remedies in the Treatment of Ununited Fractures

**Published:** 1872-05

**Authors:** A. W. Calhoun

**Affiliations:** Berlin, Prussia


### FOREIGN CORRESPONDENCE.

### NEW REMEDIES IN THE TREATMENT OF UNUNITED FRACTURES.

BY A. W. CALHOUN, M.D.

Nothing can be more harrassing to a surgeon than to find, after several months of continual treatment of a fractured limb, that union has failed to take place, and that upon each removal of the bandages, etc., the opposing ends of the bones remain almost as movable as on the day of the fracture. Such cases, however, are by no means of seldom occurrence, and may be considered, so to speak, as one of the opprobria of surgery, for frequently they stubbornly resist every effort at a cure, in spite of the many noted remedies recommended for their relief.

Amongst the many other surgical affections filling the various hospitals of this city during the last few winter months, *this* has occupied no unimportant position, and called forth no little attention and study on the part of some of the first surgeons in Berlin, and by means of phosphorus and iodine, their efforts have been encouragingly rewarded. The treatment has as yet been both internal and external, combining the two remedies, each of which is reputed to effect the same desirable end, even when used separately, but when conjoined, are thought to make assurances doubly sure.

Dr. Wegner, the chief assistant to Prof. Virchow, has for several years past been experimenting upon various animals with phosphorus, with the view of determining its bone-producing capabilities, and rather recently communicated the results of his studies and experiments to the profession through the Berlin Medical Association. In a most interesting and learned article upon this same subject, read before the Prussian Surgical Congress, assembled here a few days ago, he treated the matter more fully, and brought before the Association a large number of anatomical and other preparations or specimens, displaying the effect of phosphorus in causing a quite closely-knitted and compact new growth of bone. In some instances, some portions of bone were laid bare of its periosteum, and the animals exposed to the fumes of phosphorus, for a certain length of time, on several occasions. Diffuse periostitis was set up, and after different intervals of from four to eight weeks, various-sized protuberances of caseous and ossifying substance, of more or less thick bony consistence, were found to occupy or fill the locality previously exposed. Some of the animals thus experimented upon had decayed and hollow teeth, through which the phosphorus fumes came in contact with the alveolar processes of the jaws; and invariably, in these instances also, the same ossifying masses formed at the seat of contact as at the points uncovered by periosteum. Where the phosphorus was given internally, the normal epiphyses of the long bones, with the adjoining spongy portion of the canals, were converted into dense, hard bone. In fowls, the canals were entirely filled with new-formed solid mass, having been gradually narrowed by the concentric layers of osseous deposit, until finally the whole shaft became a firm, compact bone. Also, where limbs were fractured previous to the administration internally, the fractures were made to heal much more rapidly than when left alone with the ordinary treatment, with an abnormally dense ivory callus surrounding the united ends. The same effect resulted after resections, and after transplantations of periosteum.

In short, these experiments prove that the soft or spongy portions of bones can be made firm and solid, and that the original bone can be stimulated to a further deposit of somewhat similar substance, by infinitely small or homœopathic doses of the pure phosphorus, when continued for a sufficient time. These facts are of great practical importance, and the clinical and hospital surgeons in particular have not been slow in availing themselves of the many promising and valuable suggestions deduced from these experiments; and in accordance with the recommendations of Dr. Wegner, preparations of phosphorous are now being largely administered in resections and fractures, especially where the age of the individual, or any constitutional defect or derangement, is apt to retard or in any way interfere with the proper healing process. So far as can be judged, cures have been much hastened by this treatment.

But not until within a short time has phosphorus been prescribed with the view of bringing about the union of ununited fractures, upon which the ordinary modes of treatment and medicines have worked no good. As a rule, however, at the same time with the internal use of phosphorous in these affections, there has been locally applied, over the position of the fracture, the strong tincture of iodine, about which I may say a few words before I report the history of one case, a type of several others managed in the same manner, and with equally good terminations.

As is known, the painting of tincture of iodine over any portion of the skin sets up subcutaneous irritation or inflammation, extending to greater or less depth into the parts. But as to how deep these changes extend, or the exact nature of them, no great number of opportunities of testing have been afforded. Still, the investigations and dissections made, comparatively recently, by Dr. Schede, of "Halle," show that the parts over and upon which the iodine had been penciled for some time, and at stated intervals, were acted upon in a very similar manner as when the actual cautery had been lightly or superficially applied; and in some instances, this inflammatory action passed almost entirely through the limb, seizing upon bone as well as soft part.

A knowledge of these facts has induced the use of iodine in procuring solid growth between the ends of fractured bones, which, from some cause, have remained ununited. Prof. Langenbeck reports, that in bones lying superficially—such as the clavicle, etc.—he has succeeded in healing fractures by the local use of iodine, where all other efforts and means had disappointed him. But, as in all other diseases, and with all other remedies, there are many cases which refuse a cure from this treatment also; and hence the idea of conjoining the two articles, each well-reputed, and which, by their united effects, mostly work a favorable influence.

Early last winter (1871), came H. S——, aged fifty-five, into the clinic of Prof. Langenbeck, having fallen, a day or two previous, and fractured the humerus at the junction of the upper and middle thirds. The fracture was of a simple nature, and after bringing the ends of the bones in apposition, the plaster of Paris bandage was applied over the entire limb and the corresponding half of the thorax. The patient was otherwise strong and healthy, and consequently needed no internal or constitutional treatment.

The case progressed apparently well, and at the expiration of six weeks, the bandage was removed; but the fracture was not only unhealed, but, with the exception of pain and crepitation in the parts, the limb presented the same appearances, and, in fact, was almost as far from cure as upon the day of the accident. From this time on, till the last of February, 1872, efforts were made to effect union through innumerable local and constitutional means, but without benefit.

February 27th, 1872, commenced the daily internal use of calcaria hypophosphorica, in six grain doses morning and evening, and the local application of tincture of iodine, painting it every second day over and around the seat of the fracture.

The above, with a simple cloth bandage over the limb, constituted the treatment, which was persevered in till March 27th, at which time the fracture was firmly united—the two ends of the bone being, as it were, encased in a somewhat circular deposit or outgrowth of bone. The use of the iodine was ceased, but the preparation of phosphorus recommended to be continued for a few weeks longer, in doses of three grains twice daily, and the patient discharged as cured.

This is one of several cases treated in the same manner, and with similar results; but how much of the cure is due to the one remedy or the other, or whether the curative powers of one were more decided or predominant than the other, is of course difficult to determine, since both were used simultaneously. Separately, in the treatment of resections and fractures of the larger bones in man, neither has as yet, to any great extent, been tested; but the results of the experiments with phosphorus upon the lower animals, and the knowledge of the effect of iodine upon the human tissues, leads us to hope for much good from each in itself, and when combined, to expect, with a degree of certainty, satisfactory benefits.

At any rate, the before-mentioned experiments, with the assurance of happy results in the several similarly-managed cases observed by myself, may stimulate some one to a further investigation of the subject, which, it is reasonably to be hoped, may redound to the advancement of science and to the good of a certain class of unfortunates.

BERLIN, PRUSSIA, April 24th, 1872.

